# Contribution of the grain size QTL *GS3* to yield properties and physiological nitrogen-use efficiency in the large-grain rice cultivar ‘Akita 63’

**DOI:** 10.1270/jsbbs.21043

**Published:** 2022-03-08

**Authors:** Mitsuhiro Obara, Yoshihiro Kaneta, Ikuko Kodama, Sinichi Matsumoto, Tomohiko Kawamoto, Keiki Ishiyama, Tadahiko Mae, Amane Makino

**Affiliations:** 1 Biological Resources and Post-harvest Division, Japan International Research Center for Agricultural Sciences, 1-1 Ohwashi, Tsukuba, Ibaraki 305-8686, Japan; 2 Faculty of Bioresource Sciences, Akita Prefectural University, Shimosinjyou, Akita 010-0195, Japan; 3 Akita Prefectural Agricultural Experiment Station, Aikawa Yuwamachi, Kawabe, Akita 010-1231, Japan; 4 Graduate School of Agricultural Science, Tohoku University, 468-1 Aramaki-Aoba, Aoba-ku, Sendai, Miyagi 980-8572, Japan

**Keywords:** gran size, near-isogenic line, nitrogen-use efficiency, quantitative trait locus/loci, rice (*Oryza sativa* L.)

## Abstract

The development of crop varieties with high nitrogen-use efficiency (NUE) is thought to be important in achieving sustainable cereal crop production. The high yield large-grain rice cultivar *Oryza sativa* L. ‘Akita 63’ (*temperate* *japonica*) has high physiological NUE (PNUE) for grain yield (GY). Our previous study revealed that a large-grain allele of *GS3* is present in ‘Akita 63’. Here, we verified the influence of *GS3* on the yield properties and PNUE for GY in ‘Akita 63’. The frequency distribution of brown rice length in F_2_ crosses of ‘Iwate 75’ and ‘Akita 63’ showed a continuous distribution that could be explained by *GS3*. A near-isogenic line was developed to substitute the *GS3* segment of ‘Koshihikari’, which harbours a normal-sized grain allele, in the genetic background of ‘Akita 63’ and the line was designated as Akita63NIL*GS3*-Koshihikari. Compared with Akita63NIL*GS3*-Koshihikari, ‘Akita 63’ exhibited a significantly increased grain length, single brown grain weight and GY, although no significant differences were observed in the nitrogen content and above-ground biomass per unit of cultivated area. These results indicate that the *GS3* large-grain allele is a contributing factor to high PNUE for GY in ‘Akita 63’. These findings will facilitate the development of nitrogen-efficient rice varieties.

## Introduction

By 2050, up to 50% more food, feed and biofuel, compared with those produced in 2013, will need to be produced worldwide to meet the needs of the world population that is reaching nearly 10 billion (FAO 2017). Improvements in yield will be required to reduce the number of people who may face undernourishment in developing countries. The most efficient means of achieving this production rate is to use nitrogen-efficient crop varieties (Rosegrant *et al.* 2014). Excess nitrogen fertiliser has been utilised to obtain higher grain yield (GY) in the current rice cultivation practices (Omara *et al.* 2019). Nitrogen uptake by rice plants results in plant growth such as increased tiller number at the vegetative stage, increased spikelet number at the early phase of panicle formation, and increased grain filling at the grain filling stage (Mae 1997). Rice is the staple food for two-thirds of the world’s population. Therefore, to enhance global food security, it is important to improve the physiological nitrogen-use efficiency (PNUE), especially for GY, which is defined as the ratio of GY to the amount of nitrogen absorbed by the plants.

The large-grain *temperate japonica* cultivar *Oryza sativa* L. ‘Akita 63’ is high-yielding, with high PNUE compared with three other *temperate* *japonica* cultivars: the high-yielding cultivar ‘Yukigesyou’, old cultivar ‘Toyonishiki’ and leading Japanese cultivar ‘Akitakomachi’ (Mae *et al.* 2006). Physiological analyses have demonstrated that the large-grain size of ‘Akita 63’ contributes to the high PNUE for GY in paddy fields, although other traits also have an effect. Subsequently, Makino *et al.* (2020) compared ‘Akita 63’ with its parental lines ‘Oochikara’ (a large-grain cultivar) and ‘Akita 39’ (a normal-sized grain cultivar). They found that the large-grain size of ‘Akita 63’ is a major determinant of high PNUE for GY, especially when compared with ‘Akita 39’. Furthermore, through comparative sequencing analyses of major causal genes for four grain size quantitative trait loci (QTL), *GS3*, *GW2* (Song *et al.* 2007), *TGW6* (Ishimaru *et al.* 2013) and *qSW6* (Shomura *et al.* 2008), ‘Akita 63’ was found to contain a large-grain allele of *GS3* (Fan *et al.* 2006) that originated from ‘Oochikara’.

*GS3* was identified as a major QTL for the length and weight of grain with minor effects on grain width and thickness in a population developed from a cross between *indica* varieties (Fan *et al.* 2006). A single nucleotide polymorphism (SNP) of Os03g0407400, comprising a switch from C to A in the second exon, which produces a stop codon and induces subsequent truncation of the GS3 protein, has been demonstrated to be a functional nucleotide polymorphism for large-grain size through genetic and comparative sequencing analyses (Fan *et al.* 2006) and complementation tests (Takano-Kai *et al.* 2009). A great deal of information on *GS3* and its involvement in molecular mechanisms has been accumulated in relation to its evolution by natural variation and through the artificial selection of *Oryza* species and *Oryza* *sativa* sub-species (Lu *et al.* 2013, Mao *et al.* 2010, Takano-Kai *et al.* 2009, 2011, Yan *et al.* 2011). However, the contribution of *GS3* to yield properties and PNUE for GY remains unclear. For example, the presence of the large-grain allele of *GS3* in the large-grain ‘IR 64’ indicated the positive effect of this allele on grain weight in the genetic background of ‘Koshihikari’ (normal grain *temperate japonica* cultivar) but did not show any positive effect on GY (Ujiie *et al.* 2016). Thus, further verification studies are required to confirm the contribution of *GS3* to high yield with high PNUE in ‘Akita 63’.

In this study, we developed a near-isogenic line (NIL) with *GS3* substituted for a normal-sized grain allele in the genetic background of ‘Akita 63, which harbours a large-grain allele. We were able to verify the influence of *GS3* on yield properties and PNUE for GY in this rice cultivar.

## Materials and Methods

### Plant materials

To verify the contribution of *GS3* to the length, width and weight of brown rice in Akita 63, F_2_ plants were developed from a cross between *Oryza sativa* L. ‘Iwate 75’ (*temperate japonica*), which produces normal-sized rice grains, and ‘Akita 63’. ‘Akita 63’ was crossed with *Oryza sativa* L. ‘Koshihikari’ (*temperate japonica*), which produces normal-sized rice grains, as the pollen parent to develop a NIL in the genetic background of ‘Akita 63’. The resulting F_1_ plant was backcrossed five times with ‘Akita 63’ using a DNA marker survey across the whole genome. Selected BC_5_F_1_ plants were self-pollinated to obtain BC_5_F_2_ progeny. A target segment of *GS3* from ‘Koshihikari’ in the genetic background of ‘Akita 63’ was introduced to selected BC_5_F_2_ plants. The resulting NIL was designated as Akita63NIL*GS3*-Koshihikari. The resulting BC_5_F_3_ progeny were utilised to verify and characterise the effect of *GS3* on the size and weight of brown grains, agronomical traits, yield properties, nitrogen content and PNUE.

### Growth conditions in paddy fields

Seeds were soaked for a day at room temperature and sown in culture soil. The 28-day-old seedlings were transplanted into paddy fields. The F_2_ plants and their parental lines were transplanted into a paddy field in Sendai, Miyagi, Japan, at a density of 22.2 plants/m^2^. These plants were given 2.4 g of nitrogen fertiliser/m^2^ in the form of chemical fertiliser (N, 14%; P_2_O_5_, 14%; K_2_O, 14%). The BC_5_F_3_ plants and ‘Akita 63’ specimens were transplanted into a paddy field in Tsukuba, Ibaraki, Japan, at a density of 20.7 plants/m^2^. These plants were given 4.8 g of nitrogen fertiliser/m^2^ in the form of organic fertiliser (N, 6%; P_2_O_5_, 8%; K_2_O, 4%).

### Evaluation of the size and weight of grains and brown rice

A total of 12 grains distributed along the branch were harvested at the mature stage. Subsequently, they were dehusked using tweezers to prepare brown rice. The sizes of the grains and brown rice were evaluated using a Grain Scanner 2 (RSQI 10A, SATAKE, Hiroshima, Japan). After evaluating these traits, the grains and brown rice were dried for at least 5 days at 70°C and then weighed using an electronic balance.

### Evaluation of the agronomic traits and yield properties

A total of seven traits, namely days from sowing to heading (DTH), plant height (PH) at the harvesting stage, total dry weight of the above-ground biomass per unit of cultivated area at harvest, GY per unit cultivated area, thousand-grain weight (TGW), total grain number (TGN) and seed fertility, were evaluated in two lines, ‘Akita 63’ and Akita63NIL*GS3*-Koshihikari. The DTH values were defined as the days after sowing until the heading stage when 50% of the plants were exerting panicles in each line. The plants were harvested at the mature stage and then dried for 2 months in a well-ventilated room in the presence of an electronic fan. Six plants from each line were subject to evaluation. PH was measured using a ruler. The total dry weight of the above-ground biomass was measured, and then the material was divided into two portions, one containing the panicle and the other containing the culm and leaf. These two samples were then weighed. One panicle from each plant was weighed, and then the number of fertile and sterile gains was counted according to the standard evaluation system for rice (International Rice Research Institute 2002). The TGN was estimated by multiplying the grain number from the panicle with the ratio of total panicle weight to the panicle weight. GY was determined as the weight of winnowed rough rice, which was separated using air flow (using adjustment A for level 7 and B for level 4.5, model FV-459, Fujiwara Scientific Company Co., Ltd., Tokyo, Japan) to remove inferior grains and husks. TGW was estimated as the weight of 1,000 winnowed grains.

### Evaluation of nitrogen content

The nitrogen content was evaluated using an automatic highly sensitive NC analyser (SUMIGRAPH model NC-220F, Sumika Chemical Analysis Service, Ltd., Tokyo, Japan). After dividing the plant materials into panicles and other parts, they were oven-dried for at least 5 days at 70°C and then weighed. The dried plant materials were cut into 2–3-cm-long fragments using scissors and then ground into a powder using a rotary mill (model CT293, Verder Scientific Co., Ltd., Tokyo, Japan). After further drying, approximately 60 mg of the plant powder was weighed precisely and subjected to evaluation in accordance with the manufacturer’s protocol. PNUE for above-ground biomass was defined as the ratio of biomass to the amount of nitrogen absorbed by the plants. PNUE for GY was defined as the ratio of GY to the amount of nitrogen absorbed by the plants.

### Genotypic analysis to identify the *GS3* allele

DNA was extracted using the isopropanol precipitation method, as previously described (Obara *et al.* 2014). Fragments harbouring the functional nucleotide polymorphism of *GS3* were amplified from genomic DNA templates using PrimeSTAR HS DNA polymerase (TAKARA Bio Inc., Siga, Japan) in accordance with the manufacturer’s protocol and the following pair of primers: 5ʹ-CAGTGGCTGCGTCACTCATT-3ʹ and 5ʹ-GCTCTTACGGGAGGACATGC-3ʹ. The amplified fragments were digested with *Pst* I (TAKARA Bio Inc., Siga, Japan) in accordance with the manufacturer’s protocol. The detection of digested fragments was performed as described by Obara *et al.* (2014).

### Genotypic analyses to develop Akita63NIL*GS3*-Koshihikari

DNA was extracted for the genotypic analyses based on simple sequence repeats (SSRs) using isopropanol precipitation methods as previously described (Obara *et al.* 2014). A total of 833 SSR markers were utilised to detect polymorphisms between the appropriate ‘Akita 63’ and ‘Koshihikari’ (Obara *et al.* 2019). The markers were randomly selected from across the whole genome from those previously reported by McCouch *et al.* (2002) and the International Rice Genome Sequencing Project (2005). An additional 32 SSR markers were used to identify polymorphisms in a region close to *GS3* (Supplemental Table 1). For the SNP-based genotypic analysis, DNA was extracted using the DNeasy Plant Kit (QIAGEN, Tokyo, Japan) in accordance with the manufacturer’s protocol. The DNA was eluted with distilled water, and its concentration was determined using a NanoDrop 2000 spectrophotometer (Thermo Fisher Scientific, USA). Genotypic analysis using Kompetitive Allele Specific PCR (KASP) markers was conducted by a KASP genotyping service (LGC, Middlesex, UK). The KASP genotyping system based on SNPs has previously been described (Pariasca-Tanaka *et al.* 2015). Among the 2,015 KASP markers established by Pariasca-Tanaka *et al.* (2015), we selected 1,468 that demonstrated polymorphisms between at least three combinations of four varieties: ‘CG14’ and ‘TOG5683’ belonging to *Oryza glaberrima* Steud., O ‘IR64’ (*indica*) and WAB56-104 (*tropical japonica*).

### Statistical analyses

The data were analysed using unpaired *t*-tests (two-tailed) and a multi-regression analysis using BellCurve for Excel (Social Survey Research Information Co., Ltd., Tokyo, Japan).

## Results

### The effect of grain size on the grain weight of F_2_ plants: A multiple linear regression analysis

A multiple linear regression analysis was conducted to predict the single-brown-rice weight based on two variables, namely, length and width, of the brown rice in the F_2_ population. A significant regression equation was found [F(2, 150) = 4347, p < 0.001], with an *R*^2^ of 0.9830 and corrected *R*^2^ of 0.9828. The equation used for the multiple linear regression analysis of the brown rice weight was as follows: (single-brown-rice weight) = –24.7 + 54.1 (length) + 17.2 (width) (Table 1). The significance of the regression coefficient was 54.1 for the length and 17.2 for the width, indicating that length was a major predictor of the single-brown-rice weight.

### Distributions of size and weight of brown rice in F_2_ plants classified by the *GS3* allele

**Fig. 1.**
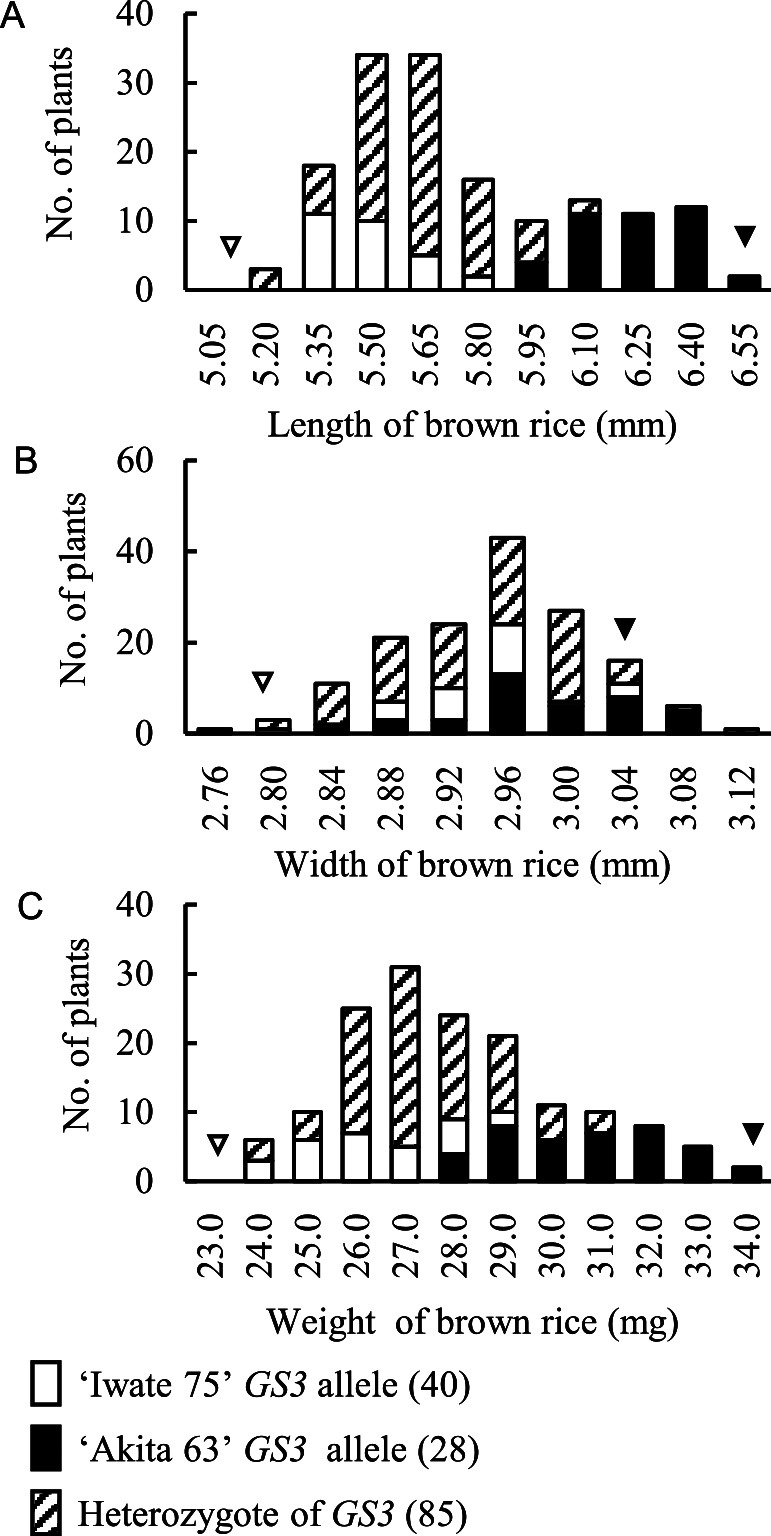
The frequency distribution of the length (A), width (B) and weight (C) of brown rice for the three genotype classes of *GS3*: ‘Iwate 75’ allele (opened column), ‘Akita 63’ allele (closed column) and heterozygote (shaded column) F_2_ plants. We used 153 F_2_ plants developed from a cross between ‘Iwate 75’, which produces normal-sized rice grains, and ‘Akita 63’. The numbers in the parentheses represent the number of F_2_ plants in each genotype.

Our previous study revealed that the large-grain allele of *GS3* is present in ‘Akita 63’ (Makino *et al.* 2020). Genotyping results identified three genotype classes: 40 F_2_ plants for ‘Iwate 75’ *GS3* allele, 28 F_2_ plants for ‘Akita 63’ *GS3* allele and 85 F_2_ plants for heterozygotes of *GS3*, among which 153 F_2_ plants were developed by crossing ‘Iwate 75’ and ‘Akita 63’ (Fig. 1). The lengths of the brown rice from the F_2_ plants were widely distributed, ranging from 5.25–6.61 mm (Fig. 1A). The distribution of brown rice length was clearly distinct between *GS3* genotype groups long-grain allele (‘Akita 63’) and normal-sized grain allele (‘Iwate 75’). In terms of width, transgressive segregation in the negative and positive directions was observed (Fig. 1B). The values of the brown rice widths from the F_2_ plants were also continuously distributed, ranging from 2.79–3.15 mm. Unlike the lengths of the genotypes, the width of each genotype was distributed in roughly the same range. The values of the brown rice weight from the F_2_ plants were widely distributed, ranging from 24.20–34.19 mg (Fig. 1C). The distribution of brown rice weight was roughly distinct between *GS3* genotype groups long-grain allele (‘Akita 63’) and normal-sized grain allele (‘Iwate 75’).

### Development of a NIL of ‘Koshihikari’ *GS3* against the genetic background of ‘Akita 63’

**Fig. 2.**
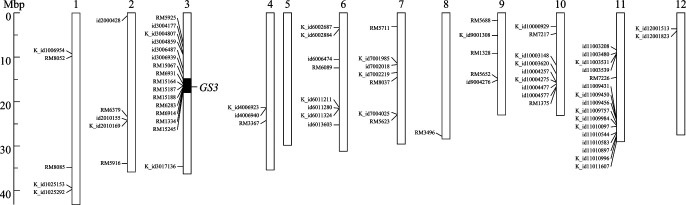
The graphical genotype of Akita63NIL*GS3*-Koshihikari. The open bars indicate the chromosomes on a physical map of the ‘Nipponbare’ sequence. The open and closed columns represent the ‘Akita 63’ and ‘Koshihikari’ segments, respectively. The straight and oblique lines represent the positions of the simple sequence repeat and Kompetitive Allele Specific PCR (KASP) markers used in this study.

We developed a NIL of ‘Koshihikari’ *GS3* for further verification in the genetic background of ‘Akita 63’ and designated it as Akita63NIL*GS3*-Koshihikari. The nucleotide substitution from C to A at 168 bp was also observed in ‘Koshihikari’ and was the same as that observed in ‘Iwate 75’ (Supplemental Fig. 1). 26 out of 865 SSR markers exhibited polymorphisms between ‘Koshihikari’ and ‘Akita 63’ (Fig. 2). Moreover, 52 of 1,468 KASP markers were polymorphic between these two lines. A total of 78 markers were utilised to develop the NIL with marker-assisted selection across the whole genome (except for chromosome 5). Two BC_5_F_2_ plants with the ‘Akita 63’ background were selected for substitution of a segment in the vicinity of *GS3* on chromosome 3 from ‘Koshihikari’. The substituted region in the NIL was at least 2.67 Mbp long and corresponded with RM15067 to RM15245 on chromosome 3.

### Effect of ‘Akita 63’ *GS3* allele on the weight and size of grain and brown rice in the genetic background of ‘Akita 63’

The effects of *GS3* allele from ‘Akita 63’ on the size and weight of grain and brown rice in the genetic background of ‘Akita 63’ were verified by growing ‘Akita 63’ and Akita63NIL*GS3*-Koshihikari in a paddy field and by obtaining accurate measurements (Table 2). Significant differences in the weights and lengths of the grain and brown rice were noted, although no differences were observed in the grain width values. Compared with Akita63NIL*GS3*-Koshihikari, the values for single-grain weights and grain lengths in ‘Akita 63’ were 14% and 9% higher, respectively. In addition, ‘Akita 63’ exhibited 14% and 13% higher values for single-brown-rice weight and brown rice length, respectively. We used two independent BC_5_F_2_ plants with the genetic background of ‘Akita 63’ to develop the NIL. Similar tendencies in these traits, except for the widths of the grain and brown rice, between these lines were also observed in the other independent line (Supplemental Table 3). Opposite changes in the widths of the grain and brown rice were observed in the NILs, although there were no significant differences in the traits between ‘Akita 63’ and Akita63NIL*GS3*-Koshihikari (Table 2, Supplemental Table 3).

### Effect of ‘Akita 63’ *GS3* allele on agronomic traits and yield properties against the genetic background of ‘Akita 63’

Two agronomic traits and the GY and its components were evaluated to estimate the effects of the ‘Akita 63’ *GS3* allele in the genetic background of ‘Akita 63’ (Table 3). Significant differences were found in GY and TGW. However, there were no significant differences in the other four traits between these lines. ‘Akita 63’ demonstrated 18% and 22% higher GY and TGW than Akita63NIL*GS3*-Koshihikari, respectively. We developed two independent BC_5_F_2_ plants with the genetic background of ‘Akita 63’ as the NIL. Similar tendencies were observed in the other independent line (Supplemental Table 4).

### The effect of ‘Akita 63’ *GS3* allele on biomass production, nitrogen content and PNUE

Our previous studies demonstrated that the large-grain size of ‘Akita 63’ was a major determinant of high PNUE for GY (Mae *et al.* 2006, Makino *et al.* 2020). The above-ground biomass, nitrogen content and HI were evaluated to estimate the effects of the ‘Akita 63’ *GS3* allele on PNUE for biomass and GY against the genetic background of ‘Akita 63’. Compared with Akita63NIL*GS3*-Koshihikari, ‘Akita 63’ exhibited a 14%, 12% and 17% higher HI, the ratio of panicle-nitrogen to plant-nitrogen and PNUE for GY, respectively (Table 4). No significant differences were observed between ‘Akita 63’ and Akita63NIL*GS3*-Koshihikari in above-ground biomass production, nitrogen content or PNUE of the above-ground biomass. We developed two independent BC_5_F_2_ plants with the genetic background of ‘Akita 63’ as the NIL. Similar tendencies were observed in the other independent NIL (Supplemental Table 5).

## Discussion

Our previous studies indicated that the large-grain size of ‘Akita 63’ is a major determinant of high PNUE for GY (Mae *et al.* 2006, Makino *et al.* 2020). Here, we determined that the length of brown rice is a major factor affecting the grain weight in the F_2_ populations (Table 1). This finding strongly indicates that grain length is a candidate trait for high PNUE for the GY of ‘Akita 63’.

Our previous study revealed that the large-grain allele of *GS3* originating from ‘Oochikara’ is present in ‘Akita 63’ (Makino *et al.* 2020). The long-grain allele of *GS3* has been found as a factor in determining the large-grain length in the rice cultivar ‘Akita 63’, demonstrating that the frequency distribution of the brown rice length in F_2_ crosses of ‘Iwate 75’ and ‘Akita 63’ showed continuous distribution that could be explained by *GS3*. (Fig. 1A). This long-grain allele of *GS3* found in large-grain varieties is heavily distributed in *indica* rice and less so in *temperate japonica* rice (Takano-Kai *et al.* 2009). In fact, ‘Iwate 75’ possesses normal-sized grains and has the normal-grain allele of *GS3* identical to ‘Koshihikari’ and ‘Nipponbare’ (Supplemental Fig. 1). From the segregation of the brown rice length depending on *GS3* alleles and comparative sequencing analyses, we concluded that *GS3* is involved in the enhancement of grain length at least in ‘Akita 63’ and that the nucleotide substitution from C to A in the 2nd exon of Os03g0407400 is a critical polymorphism for grain length between ‘Akita 63’ and ‘Iwate 75’ and ‘Akita 63’ and ‘Koshihikari’.

There may be another QTL for brown rice size in ‘Akita 63’. At least 43 genes responsible for controlling the grain size via cell proliferation and expansion of the spikelet hull, were identified (Li *et al.* 2018). Three QTL for grain length—including *GS3*, two QTL for grain width and three QTL for grain thickness—were detected while mapping populations derived from the large-grain cultivar ‘Cytoto’ (Kato *et al.* 2011). Furthermore, the ‘Cytoto’ allele at the QTL for grain length had a positive effect on the grain lengths. The multiple insertions of positive genes for grain size suggest the possibility that ‘Akita 63’ harbours another QTL for the length and width of brown rice. In fact, a wide distribution of brown rice lengths was noted in each group for the long-grain allele, ranging from 5.97 to 6.61 mm, and for the normal-sized grain allele, ranging from 5.36 to 5.87 mm (Fig. 1A). In terms of the brown rice width, it was difficult to explain the distribution with the *GS3* allele alone (Fig. 1B). QTL mapping with high density linkage maps using next generation techniques will enable the detection of almost all QTL for grain size. However, the segregation of the brown rice lengths was distinct between *GS3* genotype groups [long-grain allele (‘Akita 63’) and normal-sized grain allele (‘Iwate 75’), Fig. 1]. Thus, further analyses involving Akita63NIL*GS3*-Koshihikari may be an adequate approach to verifying the contribution of *GS3* from ‘Akita 63’ to yield properties and PNUE for GY.

‘Akita 63’ exhibited significantly higher values for the length and weight of brown rice and grain but no differences in the widths of brown rice or grain compared with Akita63NIL*GS3*-Koshihikari (Table 2, Supplemental Table 3). As previously reported, a significantly lower grain length was observed in two independent transgenic plants, which had been introduced with a normal-sized grain allele, designated as C-allele, into an AIS22 substituted by a large-grain allele from ‘IR 24’ with the genetic background of ‘Asominori’ (*temperate japonica*, normal-sized grain) (Takano-Kai *et al.* 2009). This evidence strongly supports the contribution of the long-grain allele of *GS3* to grain length in ‘Akita 63’. However, an increase in the grain width by introducing the normal-grain allele of *GS3* into AIS22 was not observed between ‘Akita 63’ and Akita63NIL*GS3*-Koshihikari, as there was no significant difference in the grain width (Table 2, Supplemental Table 3). This discrepancy might be due to the different control of the genetic backgrounds. The grain widths for AIS22 and ‘Akita 63’ were 2.9 (Takano-Kai *et al.* 2009) and 3.32 mm (Table 2), respectively.

This variation in the *GS3* allele may lead to the high GY of ‘Akita 63’. High GY was found to be the result of an increased grain length and corresponding increase in grain weight, which leads to a high harvest index. A higher GY can be achieved either by increasing the biomass produced per unit of cultivated area, increasing the harvest index or both (Yoshida 1981). Compared with Akita63NIL*GS3*-Koshihikari, ‘Akita 63’ exhibited a significant increase in TGW for yield components (Table 3, Supplemental Table 2). The increase in GY could almost be explained by the TGW. The remaining yield components, TGN per unit cultivated area and seed fertility, were not significantly different between ‘Akita 63’ and Akita63NIL*GS3*-Koshihikari. In addition, the above-ground biomass, including the panicle, leaves and straw, was not different between these lines (Table 4, Supplemental Table 5). However, our findings did not concur with the previous research involving chromosome substitution segment lines with the genetic background of *temperate japonica* rice (Ujiie *et al.* 2016). In that report, the positive effect of the large-grain allele of *GS3* was observed for the TGW and HI in the genetic background of ‘Koshihikari’ (normal-sized grain cultivar), but no effect on GY was observed. This discrepancy may be due to the differences in the calculation of GY. While Ujiie *et al.* (2016) estimated GY from the yield components, in the present study, GY was directly determined.

Our NIL experiments indicated the contribution of the large-grain allele of *GS3* to the PNUE for GY but not biomass production in the genetic background of ‘Akita 63’. As aforementioned, we assume that the increase in grain length induced high PNUE in ‘Akita 63’. Compared with Akita63NIL*GS3*-Koshihikari, ‘Akita 63’ exhibited a significantly higher ratio of panicle-nitrogen to plant-nitrogen and PNUE for GY (Table 4, Supplemental Table 5). However, the values for nitrogen uptake and grain number were similar between ‘Akita 63’ and Akita63NIL*GS3*-Koshihikari, as there were no significant differences in the nitrogen content and TGN per unit of cultivated area, respectively. Those similarities and differences between ‘Akita 63’ and Akita63NIL*GS3*-Koshihikari were critical to considering the effects of the large allele of *GS3* in PNUE and GY in ‘Akita 63’. In a genetic background of *indica* cultivars, previous NIL experiments have confirmed the effects of the large allele of *GS3* on grain length, TGW and GY in plants grown in a paddy field (Mao *et al.* 2010). However, it is difficult to determine, which traits contributed to an increase in GY with increasing biomass production per unit cultivated area, harvest index or both, because Mao *et al.* (2010) did not examine biomass production.

In conclusion, we developed Akita63NIL*GS3*-Koshihikari containing the normal-sized grain size allele of *GS3* in the genetic background of the large-grain cultivar ‘Akita 63’. The agronomic and physiological experiments conducted in this study indicated that the large-grain allele of *GS3* contributes to PNUE for GY in the genetic background of ‘Akita 63’. However, the large-grain allele of *GS3* is not involved in a trade-off between grain size and grain number per unit of cultivated area in the genetic background of the large-grained ‘Akita 63’. Our findings will facilitate nitrogen-efficiency breeding in rice by modulating the grain size. Further analyses to verify and characterise the effects of the large-grain allele of *GS3* from ‘Akita 63’ in different genetic backgrounds harbouring normal-sized grain alleles of *GS3* will be initiated to facilitate precision breeding approaches to developing sustainable rice varieties. The development and characterisation of new NILs are ongoing.

## Author Contribution Statement

MO, YK, TM and AM designed the experiments; MO, IK, TK and SM developed the genetic materials; MO and YK performed most of the experiments; MO, KI, TM and AM analysed the data; MO, KI, TM and AM wrote most of the manuscript.

## Supplementary Material

Supplemental Figure

Supplemental Tables

## Figures and Tables

**Table 1.** Multiple linear regression for single-brown-rice weight in 153 F_2_ plants developed from a cross between ‘Iwate 75’ and ‘Akita 63’

Variable	Partial regression coefficient	Standard error	*t* value
Length	4.75	0.0878	54.1
Width	7.45	0.4333	17.2
Constant	–24.8	1.0032	–24.7

**Table 2.** Sizes of grain and brown rice from Akita63NIL*GS3*-Koshihikari in the genetic background of ‘Akita 63’ grown in a paddy field

Traits	‘Akita 63’	Akita63NIL*GS3*-Koshihikari	*P^c^*	Relative amount*^d^*
Single-grain weight (mg)	35.7 ± 1.0	31.2 ± 2.6	<0.01	114%
Length of grain (mm)	8.53 ± 0.17	7.83 ± 0.19	<0.001	109%
Width of grain (mm)	3.32 ± 0.08	3.28 ± 0.04	0.363	101%
Single-brown-rice weight*^a^*^,^*^b^* (mg)	30.8 ± 1.2	26.9 ± 1.1	<0.001	115%
Length of brown rice*^a^* (mm)	6.54 ± 0.09	5.80 ± 0.10	<0.001	113%
Width of brown rice*^a^* (mm)	2.83 ± 0.05	2.79 ± 0.05	0.305	101%

*^a^* Brown rice was prepared at the mature stage. The values are expressed as mean and standard deviation (SD) (n = 6). *^b^* The weight of brown rice (hulled rice) was adjusted to a moisture content of 0.14 g H_2_O g^–1^. *^c^* Significance of the differences between ‘Akita 63’ and the NIL evaluated using an unpaired *t*-test (n = 6). *^d^* Relative amount for ‘Akita 63’ based on the NIL.

**Table 3.** The agronomic traits and yield properties of Akita63NIL*GS3*-Koshihikari in the genetic background of ‘Akita 63’ grown in a paddy field

Traits*^a^*	‘Akita 63’	Akita63NIL*GS3*-Koshihikari	*P^c^*	Relative amount*^d^*
DTH (days)	92	92		100%
PH (cm)	107.1 ± 1.7	108.7 ± 5.3	0.551	99%
GY*^b^* (g/m^2^)	748 ± 45	635 ± 103	<0.05	118%
TGW*^b^* (g)	30.6 ± 1.2	25.1 ± 0.9	<0.001	122%
TGN (No./m^2^)	31,305 ± 2,463	33,585 ± 2.787	0.201	93%
Seed fertility	0.817 ± 0.034	0.762 ± 0.075	0.165	107%

*^a^* The data is expressed as mean and standard deviation (SD; n = 6). *^b^* Grains refined by wind-based selection were adjusted to a moisture content of 0.14 g H_2_O g^–1^. *^c^* Significance of the differences between ‘Akita 63’ and the NIL evaluated using an unpaired *t*-test (n = 6). *^d^* Relative amount for ‘Akita 63’ based on the NIL. *DTH*: days from sowing to heading, *PH*: plant height at the harvesting stage, *GY*: grain yield unit cultivated area, *TGW*: thousand-grain weight, *TGN*: total grain number.

**Table 4.** Nitrogen content and physiological nitrogen-use efficiency of Akita63NIL*GS3*-Koshihikari in the genetic background of ‘Akita 63’ grown in a paddy field

Trait	‘Akita 63’	Akita63NIL*GS3*-Koshihikari	*P^a^*	Relative amount*^b^*
Above-ground biomass (g/m^2^)	1,373 ± 27	1,369 ± 76	0.908	100%
HI	0.546 ± 0.023	0.481 ± 0.04	<0.05	114%
Nitrogen content (gN/m^2^)	10.14 ± 0.33	10.01 ± 0.54	0.669	101%
Ratio of panicle-N to plant-N	0.683 ± 0.018	0.608 ± 0.04	<0.01	112%
PNUE for above-ground biomass*^c^* (g/gN)	134.2 ± 5.4	135.6 ± 3.5	0.625	99%
PNUE for GY*^d^* (g/gN)	71.9 ± 3.1	61.5 ± 8.6	<0.05	117%

*^a^* Significance of the differences between ‘Akita 63’ and the NIL as assessed using an unpaired *t*-test (n = 6). *^b^* Relative amount for ‘Akita 63’ based on the NIL. *^c^* PNUE for above-ground biomass was defined as the ratio of biomass to the amount of nitrogen absorbed by the plants. *^d^* PNUE for GY was defined as the ratio of GY to the amount of nitrogen absorbed by the plants. *HI*: harvest index, *N*: nitrogen, *PNUE*: physiological nitrogen-use efficiency, *GY*: grain yield per unit cultivated area.
