# Supplementary material for: Contribution of the grain size QTL GS3 to yield properties and physiological nitrogen-use efficiency in the large-grain rice cultivar ‘Akita 63’
Source: Breed Sci. 2022 Mar 8;72(2):124–31. doi: 10.1270/jsbbs.21043 (PMC9522531; doi:10.1270/jsbbs.21043)
Supplement: Supplementary file 1 — Supplemental Figure [file 72_124_s1.pdf]

doi: 10.1270/jsbbs.21043

Lines	Sequences
‘Akita 63’	5′-GCCTCCAGATGCTGA-3′
	A S R C *
‘Iwate 75’	5′-GCCTCCAGATGCTGC-3′
‘ Koshihikari ’	A S R C C

Supplemental Fig. 1. The comparison of sequences around the functional nucleotide polymorphism in *GS3*.

A direct sequencing method was employed to obtain the GS3 sequences in ‘Iwate 75’ and ‘Akita 63’. Fragments were amplified from genomic DNA to create a template using Prime STAR HS DNA polymerase (TAKARA Bio Inc., Shiga, Japan) in accordance with the manufacturer’s protocol and six pairs of primers (Supplemental Table 2). The sequencing analyses were conducted by a DNA sequencing service (Eurofins, Tokyo, Japan) using appropriate primers (Supplemental Table 2). The *GS3* sequence from ‘Akita 63’ was registered to the DNA database of Japan as LC389068.

The sequence of *GS3* from ‘Iwate 75’ was the same as that from ‘Nipponbare’. There were four SNPs between ‘Iwate 75’ and ‘Akita 63’ (LC389068) in the *GS3* sequence. Among them, an SNP in exon 2 demonstrated a nucleotide substitution in ‘Akita 63’ of C to A at 165 bp downstream from the first methionine (Supplemental Fig. 1). Unlike ‘Iwate 75’, the substitution in ‘Akita 63’ produced a termination codon causing a premature stop in the protein.
