# Supplementary material for: Contribution of the grain size QTL GS3 to yield properties and physiological nitrogen-use efficiency in the large-grain rice cultivar ‘Akita 63’
Source: Breed Sci. 2022 Mar 8;72(2):124–31. doi: 10.1270/jsbbs.21043 (PMC9522531; doi:10.1270/jsbbs.21043)
Supplement: Supplementary file 2 — Supplemental Tables [file 72_124_s2.pdf]

doi: 10.1270/jsbbs.21043

Supplemental Table 1. SSR markers flanked to *GS3* used in this study

Locus name	Forward primer sequence (5'→3')	Reverse primer sequence (5'→3')
RM1164	TTTCTGGCGACGTGATTTGTCG	CAATTCGGAAGAGCAAACATGACC
RM5684	ATTCTGACGCACCAGTACCCTACG	CACATCGAGGATCTGATTGAACTGG
RM5684	TTCACCACATCGAGGATCTG	ATTCTGACGCACCAGTACCC
RM6146	AAAGGAGGGAGGAGGCAGTAGC	GGAACATCTTCTCCGGTTCTCC
RM6283	GATCAGGTGGTCGGTTCCTTACC	CCTGTTGGAGACTGAGCTGATGC
RM6881	CGACTGATTCGATTCCACAATGATCC	GCAGCTGGAAGCAGAGGAAGACG
RM6914	AAGAACCCACCTGCGGTTAGC	CTACAGCTTTCTTGATTCGCTTGG
RM6931	GCATTTGCTTGTACCTTCTGTTGG	AACCACACACATCCACGAGTCC
RM6931	AGTATCATGTAGTGCCACAA	CTCCACTGTAACTCGTTCTC
RM6959	GATTCCTATGGAGGATTGTTGC	AACTCCACCGGTGTTAAGAAGG
RM7431	CGGGACTTCTCATAGGCATG	ACTACTGGCTTCCCACAACG
RM15031	CTTCGAGCACAAGCACTAGATCG	CTCCAAATCCAAAGGACCAAACC
RM15032	GTGATGCTCGCCGTCATGTCC	TTTCGGCTCTGACGATGGATGG
RM15038	GTTGCAAATTAAGGGAGGGAAGG	CGTACGCAAGTACAACGGTACAGC
RM15040	CAAACAAATCGTGGATGGATGG	CGCACACACGCAAATATATAGTCC
RM15041	CTGTTTACGAGCTCCTCTCCAAATCC	GACGGTGTAGTCGTTGCTGTCG
RM15045	TTTGTCCTAGTTCCCTGCTGTTGC	AGCTTAGACAGCCCGAGAATGC
RM15046	TTCACCGCTCAAGCCAACTTCG	AGCTTGGCCAAAGGGAGAGTCG
RM15067	GGGTGACTCGGTCTTAGAATCG	GATGCACATGCATTGTCTAGGG
RM15072	AATGCTGCTGCTACCTGCTACCG	GCAGGCACGTCAAACAAATGG
RM15082	GGCTCTCTGTAGGATGGAGAAGC	GAGCTCTGAATGATGCTGACTGC
RM15087	CACCTCGTTCCTACGTCATATTGC	GGCGGCATAGTAGCGTTTATAGG
RM15089	GGACGAAGGACAACCTCGTAACC	ATCTATCAGCCCACCACACACG
RM15107	CCCTAGGGTTGTATGTTGTTGG	ACCCGTAGATAACCCATACCTAACC
RM15164	CGGAGGGAGTAGATTACAAGTCACG	ACCTTCTCTCCTTTCGCTCTTCC
RM15187	AGCCTCCTAGAACGGCAAAGG	CTATGTTTCGTGCAACCAAGTCC
RM15188	AGCCTCCTAGAACGGCAAAGG	CTATGTTTCGTGCAACCAAGTCC
RM15226	CCCTACACTACGCTCACAAACAAACG	TAGAGCGCCGATATCCCGAAGC
RM15236	CACTCCCTCCTCTCTCCTCTCC	GTTGGTTGGTCGGTTGCTTACC
RM15243	AAGATTGAAGAAGCGGTCAAGC	GCTTGCATGCATAGATTTCTCC
RM15245	AGGATTTACACGCGCTTTGAGC	CATCAACGGCAGTAGAAGGTTTCC
RM15303	GAATCGGGTCTACGGTTTAGG	AAAGGAAGAGAAGAGGCAACG

Supplemental Table 2. Primers to obtain specific fragment covering genomic fragment of *GS3* and to direct sequence analyses used in this study

Fragment[a]	A use	Primer sequence (5'→3')
1	Amplifying, sequencing	CGAACATGTTTTCTTGGCTTCT
1	Amplifying, sequencing	TGGAGGCTATATAGGGAAATCTGC
1	Sequencing	TGCAAGACCAGCGATCAAACTA
1	Sequencing	TCAGCTATAGCACGGACTCCAA
1	Sequencing	TCCACACAATCGACGAAGTTCA
2	Amplifying, sequencing	TCCCTCGTTTCAGGAGATACCA
2	Amplifying, sequencing	CGGCCATTTCGGTAGAACCTA
2	Sequencing	TGCAAGACCAGCGATCAAACTA
2	Sequencing	CGTCGAGAATTGTATATCTGATCAACC
2	Sequencing	TCACTGGTGGCAGATTACATGG
3	Amplifying, sequencing	CGTCGAGAATTGTATATCTGATCAACC
3	Amplifying, sequencing	TCTTGAGGTTGAAGGAGGAGGA
4	Amplifying, sequencing	TCCCACAAAACCATCAACTTGT
4	Amplifying, sequencing	GCTCTCGCATGAGAGCCAATAC
5	Amplifying, sequencing	GTGTGCGTCTGCATGCATTATT
5	Amplifying, sequencing	GCACAGGAAATCAAGCCAATAA
5	Sequencing	AGTTCCCCAAAAACTGCTCCTC
5	Sequencing	AGCTAAGCATTCGTGGCTTCAG
6	Amplifying, sequencing	GCGCATGCATATAGTTTCTCGTC
6	Amplifying, sequencing	TCCACACAATCGACGAAGTTCA

[a] A total of six fragments were amplified and sequenced GS3 allele of ‘Akita 63’ and ‘Iwate 75’.

Supplemental Table 3. Sizes of grain and brown rice from Akita63NIL*GS3*-Koshihikari in the genetic background of 'Akita 63' grown in a paddy field

Traits	Akita 63	Akita63NIL*GS3*-Koshihikari	P^c	Relative amount[d]
Single-grain weight (mg)	35.7±1.0	33.6±1.3	< 0.05	106%
Length of grain (mm)	8.53±0.17	7.80±0.10	< 0.001	109%
Width of grain (mm)	3.32±0.08	3.39±0.04	0.084	98%
Single-brown-rice weight[a, b] (mg)	30.8±1.2	28.1±0.6	< 0.001	110%
Length of brown rice[a] (mm)	6.54±0.09	5.88±0.06	< 0.001	111%
Width of brown rice[a] (mm)	2.83±0.05	2.83±0.04	0.943	99%

[a] Brown rice was prepared at the mature stage. The values are expressed as mean and standard deviation (SD) (n = 6).

[b] The weight of brown rice (hulled rice) was adjusted to a moisture content of 0.14 g H_2O g^{-1}.

[c] Significance of the differences between 'Akita 63'and the NIL evaluated using an unpaired *t*-test (n = 6).

[d] Relative amount for 'Akita 63' based on the NIL.

Supplemental Table 4. The agronomic traits and yield properties of Akita63NIL*GS3*-Koshihikari in the genetic background of 'Akita 63' grown in a paddy field

Traits[a]	Akita 63	Akita63NIL*GS3*-Koshihikari	*P*[c]	Relative amount[d]
DTH (days)	92	92		100%
PH (cm)	107.1±1.7	108.0±3.6	0.621	99%
GY[b] (g/ m^2)	748±45	609±118	＜0.05	123%
TGW[b] (g)	30.6±1.2	25.6±1.7	＜0.001	119%
TGN (No./ m^2)	31,305±2,463	31,793±2.887	0.791	98%
Seed fertility	0.817±0.034	0.772±0.035	0.086	106%

[a] The data is expressed as mean and standard deviation (SD; n = 6)

[b] Grains refined by wind-based selection were adjusted to a moisture content of 0.14 g H_2O g^{-1}.

[c] Significance of the differences between 'Akita 63'and the NIL evaluated using an unpaired *t*-test (n = 6).

[d] Relative amount for 'Akita 63' based on the NIL.

DTH: days from sowing to heading, *PH*: plant height at the harvesting stage, *GY*: grain yield unit cultivated area, *TGW*: thousand-grain weight, *TGN*: total grain number

Supplemental Table 5. Nitrogen content and physiological nitrogen-use efficiency of Akita63NIL*GS3*-Koshihikari in the genetic background of 'Akita 63' grown in a paddy field

Traits	Akita 63	Akita63NIL*GS3*-Koshihikari	*P*[a]	Relative amount[b]
Above-ground biomass (g/m^2)	1,373±27	1,316±55	0.065	104%
HI	0.546±0.023	0.486±0.052	<0.05	112%
Nitrogen content (gN/m^2)	10.14±0.33	9.46±0.8	0.111	107%
Ratio of panicle-N to plant-N	0.683±0.018	0.623±0.056	<0.05	110%
PNUE for above-ground biomass[c] (g/gN)	134.2±5.4	138.3±7.2	0.331	97%
PNUE for GY[d] (g/gN)	71.6±3.1	62.0±7.1	<0.05	116%

[a] Significance of the differences between 'Akita 63' and the NIL as assessed using an unpaired *t*-test (n = 6).

[b] Relative amount for 'Akita 63' based on the NIL.

[c] PNUE for above-ground biomass was defined as the ratio of biomass to the amount of nitrogen absorbed by the plants.

[d] PNUE for GY was defined as the ratio of GY to the amount of nitrogen absorbed by the plants.

HI: harvest index, *N*: nitrogen, *PNUE*: physiological nitrogen-use efficiency, *GY*: grain yield per unit cultivated area
